# The NMR Spectral Measurement Database: A System for Organizing and Accessing NMR Spectra of Therapeutic Proteins

**DOI:** 10.6028/jres.126.035

**Published:** 2021-12-16

**Authors:** Niksa Blonder, Frank Delaglio

**Affiliations:** 1 Chemical Sciences Division, Material Measurement Laboratory, National Institute of Standards and Technology, Gaithersburg, MD 20899, USA; 2 Institute of Bioscience and Biotechnology Research, National Institute of Standards and Technology and University of Maryland, Rockville, MD 20850, USA

**Keywords:** database, nuclear magnetic resonance, spectral measurement

**Software DOI:** https://doi.org/10.18434/mds2-2495

**Software Version:** 1.0

## 1 Summary

The Nuclear Magnetic Resonance Spectral Measurement Database (NMR-SMDB) was developed for the purpose of organizing and searching NMR spectral data of protein therapeutics, linking spectra to corresponding sample information and enabling quick access to full datasets and entire studies. In addition to supporting internal research at the National Institute of Standards and Technology (NIST), the system could facilitate data access to stakeholders outside of NIST, and future versions of the database software itself could be installed by others for their own data storage and retrieval.

Several groups in the NIST Biomanufacturing Program develop methods to meet the unique measurement needs of protein therapeutics, which must maintain both their chemical structures and their three-dimensional shapes without aggregating in order to be safe and effective. One general approach to this measurement challenge is spectral fingerprinting, based upon the simple idea that change in a molecular shape or chemical structure will cause changes in corresponding spectra. In this approach, confirmation of an intact structure can be revealed by the degree of similarity between a measured spectrum and reference spectra, and the specific nature and magnitude of any spectral differences can be used to characterize the corresponding change in structure [1].

The sensitivity and specificity of spectral fingerprinting depends on the analytic methods employed, and these include circular dichroism (CD), which provides low resolution information about the secondary structure composition of a protein, and mass spectrometry (MS), which provides high resolution, highly detailed information about the chemical structure of a protein, but not direct information about its three-dimensional shape. Fingerprinting by NMR spectroscopy is an ideal complement to these measurement methods, because NMR spectra are sensitive to molecular shape and interactions as well as chemical structure, and NMR can provide this information at atomic resolution. Meeting the measurement needs of protein therapeutics leads to an additional computational need: spectral data must be organized in order to develop and exploit spectral fingerprinting methods.

There are projects to develop general purpose formatting, storage, and retrieval of analytical data, including the Allotrope Foundation Data Standard and the Analytical Information Markup Language (AnIML) [2], [3]. These are large projects that are still changing rapidly, and their ontologies for NMR data are incomplete. Furthermore, regardless of the underlying format of spectral data and metadata, the practical details of data entry, including capturing information that might be contained only in written lab notebooks, will be similar. For these reasons, we were motivated to develop our own basic system for storage and retrieval of NMR data, so that it could be easily customized for our primary use cases, so that we could evaluate procedures for capturing data and metadata, and so that we could more readily determine what capabilities and features would be needed for a long-term database solution. In addition to our own applications inside NIST, a successful system could be used to post data to be shared with stakeholders outside of NIST, or the software system itself could be installed by others to manage their own data.

A key tool for developing spectral fingerprinting analytical methods is the use of forced degradation studies, where a sample is intentionally subjected to damaging conditions such as multiple cycles of freezing and thawing, or exposure to disruptive chemical agents. Such a study has source materials that are used to prepare one or more NMR samples, and each NMR sample is associated with one or more independent variables such as concentration of an additive. Each NMR sample is associated with a series of replicate NMR measurements, and each measurement is associated with a spectrum generated by Fourier reconstruction of the measured data.

Each NMR instrument vendor uses its own ad hoc data format. In a typical case, raw data from an NMR spectrometer consists of one data directory for each measurement containing measured NMR data in binary form along with parameter files detailing the instrument settings. However, there is no information about the sample, and no information indicating how related measurements are grouped. This information exists only in lab notebooks. Therefore, there is no central index of the NMR measurements being made for convenient integration and correlation of NMR measurements in order to analyze a study as a whole.

**Fig. 1:**
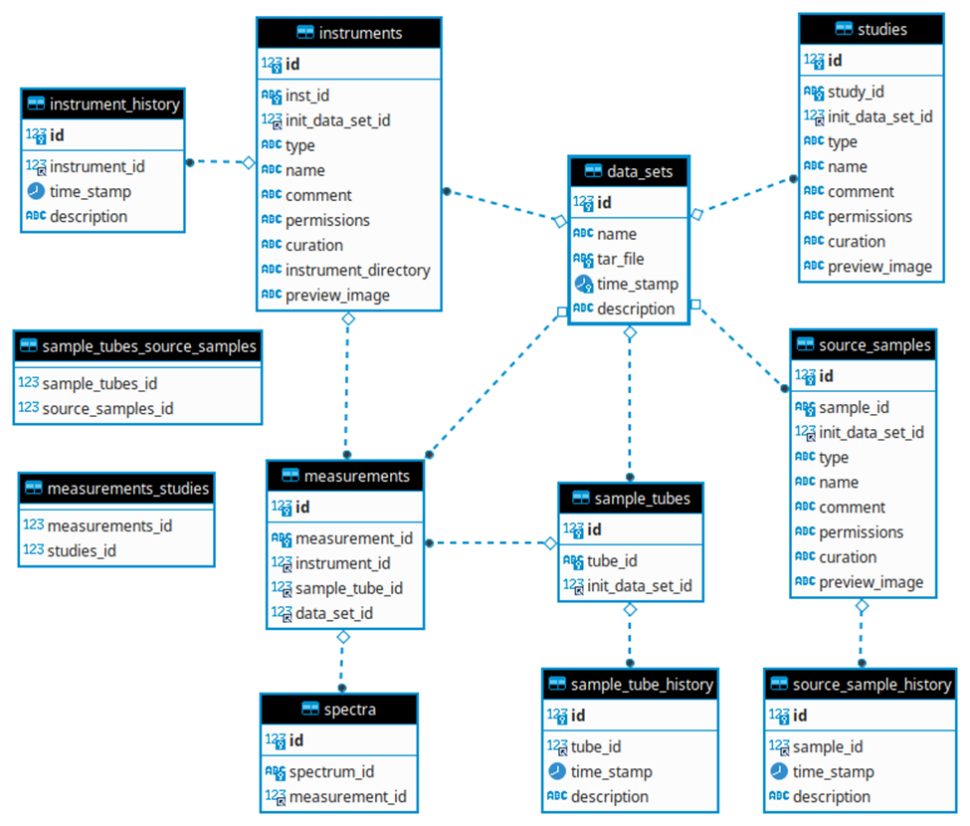
Schema for the NMR Spectral Measurement Database. The links between objects in the database are formed by one or more history entries, each with a timestamp. For example, for each measurement there is a corresponding history entry for a given instrument, documenting when the measurement was performed.

NMR-SMDB was developed to facilitate the process of linking NMR measurements to their samples by collating and organizing these measurements into studies to facilitate further downstream analyses. Furthermore, it helps to establish the protocols and practices needed for capturing metadata that would normally only be written in a lab notebook. The database schema, shown in Fig. 1, define concepts of: NMR Instrument, Source sample, NMR sample, NMR Measurement, NMR Spectrum, and Study.

The database system software provides the following features:

Packaging and upload of data as a TAR archive file

Automatic parsing of TAR archives and populating the NMR database

**Fig. 2:**
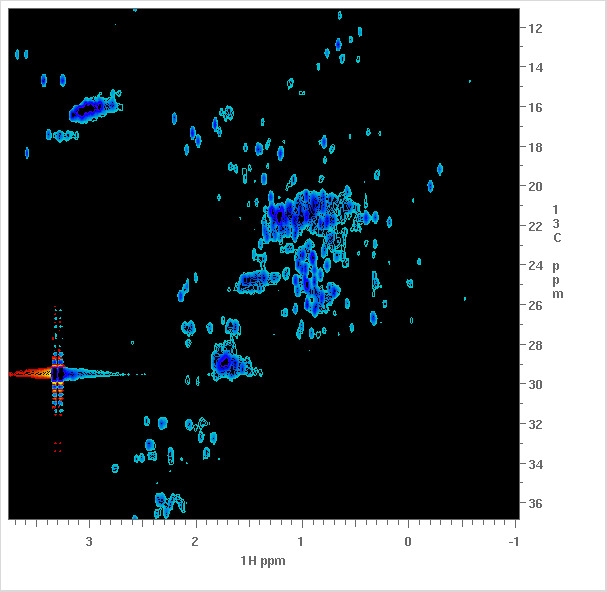

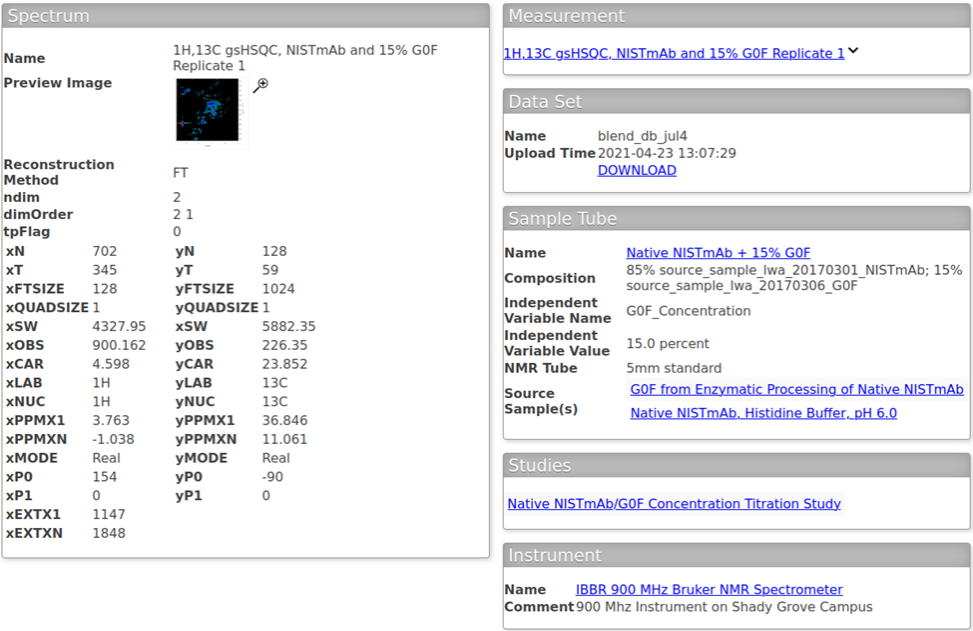
Top: An example of the Spectrum View, which lists metadata about the spectrum (left). At right, top to bottom: the parent measurement that generated the spectrum, the Data Set (TAR archive) that included the spectrum, the Sample Tube (NMR sample) that was measured, the Study (related collection of measurements) that includes the spectrum, and the NMR Instrument used to make the measurement. Bottom: the corresponding preview image of the spectral data.

Web based interface to search NMR study metadata and view measurement spectra (Fig. 2)

Retrieval of uploaded datasets

**2:** Software Specifications

**NIST Operating Unit(s)**	Material Measurement Laboratory, Chemical Sciences Division, Institute for Bioscience and Biotechnology Research (IBBR)
**Category**	Data mining
**Targeted Users**	IBBR, NMR research scientists
**Operating System(s)**	Cross-platform
**Programming Language**	Python, PHP, SQL and JavaScript
**Inputs/Outputs**	TAR archive file containing NMR raw data and metadata extracted by NMRPipe software [4]
**Documentation**	Documentation can be found at: https://www.ibbr.umd.edu/nmrpipe/permanent/nmr-smdb.pdf
**Accessibility**	N/A small-scale research tool.
**Disclaimer**	https://www.nist.gov/director/licensing

## 3 Methods

**Fig. 3:**
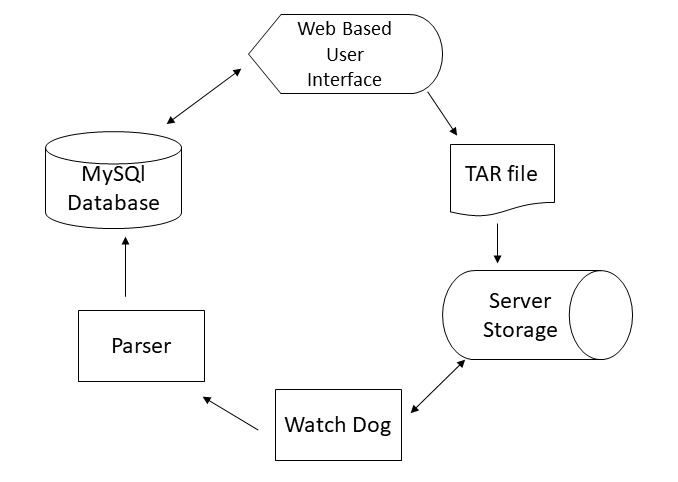
A diagram of the NMR Spectral Measurement Database software and data components.

The components of NMR-SMDB are diagrammed in Fig. 3.

### 3.1 NMR Watchdog

This is a Python daemon that continuously runs in the background and monitors upload directory of the web interface for changes [5]. The watchdog has two tasks:

Spawns NMR parser process when it detects a new upload
Logging uploads and deletions of datasets

The uploaded data is in the form of a TAR archive with one or more directories of NMR data. The directories have been populated with metadata in CSV format via special purpose C-shell and TCL scripts in the NMRPipe spectral processing system [4].

### 3.2 NMR Parser

This Python script picks a newly uploaded dataset and performs the following operations:

• Extracts only metadata and spectral data from TAR files and parses the data
• Populates/updates the NMR-SMDB database
• Checks for errors in data formats and deletes dataset and the associated TAR file should any errors occur during parsing/input of data into database
• Logs any encountered errors

### 3.3 MySQL database

MySQL was chosen as a backend database engine due to its cross-platform compatibility, Open-Source license and wide use [6]. Future development of analytics apps can also make use of the data stored in the database for statistical analysis.

### 3.4 Web Based User Interface

The end-user interface for NMR-SMDB was developed in PHP and JavaScript programming languages. NMR data can be searched and filtered by a number of different criteria such as source sample names, sample composition, independent variable name or value etc. A non-interactive visual representation of NMR spectra is also available.

In addition to performing searches the web interface allows for upload of new data sets and download of already entered datasets.

## 4 Conclusions

The NMR-SMDB is our first attempt to address a critical need within the NIST biomanufacturing program: organizing and searching NMR spectral data of protein therapeutics, and relating these spectra to corresponding sample and study information. In addition to meeting this specific need, the database system serves as a test environment for establishing practical protocols for capturing data and metadata that might exist only in hand-written lab notebooks. As such, the knowledge derived from implementing and testing this system will also benefit other analytical measurements for biopharma applications, in particular mass spectral data. To support this goal, future implementations of the NMR-SMDB could employ community-based interoperable data and schema formats, opening the way to integrated analysis of multi-modal data, and enabling effective data access for future machine learning applications.
